# Impact of the pandemic on surgical oncology in Piedmont, Italy: a retrospective observational study: Paolo Ragusa

**DOI:** 10.1093/eurpub/ckac131.079

**Published:** 2022-10-25

**Authors:** P Ragusa, G Lo Moro, M Aglietta, M Airoldi, A Comandone, C Previti, F Bert, R Siliquini

**Affiliations:** 1 Department of Public Health Sciences, University of Turin, Turin, Italy; 2 Department of Oncology, University of Turin, Turin, Italy; 3 Candiolo Cancer Institute-FPO-IRCCS, Candiolo, Italy; 4 Rete Oncologica del Piemonte e della Valle d'Aosta, Turin, Italy; 5 Oncology Unit 2, A.O.U. City of Health and Science of Turin, Turin, Italy; 6 Department of Oncology, San Giovanni Bosco Hospital, Turin, Italy; 7 Italian Group of Rare Tumors, Turin, Italy; 8 A.O.U. City of Health and Science of Turin, Turin, Italy

## Abstract

**Background:**

To prevent the spread of SARS-CoV-2, containment measures were implemented leading to huge healthcare changes worldwide. This study aimed to describe the impact of COVID-19 pandemic on surgical oncology healthcare in a large Italian sample.

**Methods:**

A retrospective observational study included 99651 patients admitted to the hospitals of Piedmont (Northern Italy) to undergo oncological surgery, provided in ordinary hospitalization. We compared data of 2020 with 2016-2019 mean values. Data were stratified by tumor site, year, month and admission way. Chi-squared tests were used to assess differences in the percentage of admission modes between 2020 and 2016-2019.

**Results:**

An overall reduction in oncological surgery (-12.3%) was observed in 2020 (n = 17923) compared to the mean of period 2016-2019 (n = 20432). A relevant decrease began in March (-11%), continued in April (-18%) and peaked in May (-26%). There was a greater reduction in surgery of breast (-19.2%), bladder (-17.5%), colorectal (-16.5%), kidney (-14.2%), prostate (-14%). Little or no difference was observed for liver (-5.2%), body of uterus (-0.54%), ovary (-0.07%), lymphoma (+4.5%). There was a marked reduction of non-emergency admissions (-13.6%), in particular for some tumor sites: colorectal (-19.4%), breast (-19.4%), bladder (-18.7%). The overall volume of surgeries following an emergency access was unchanged (-0.3%). The proportion of hospitalizations with emergency access increased (p < 0.001).

**Conclusions:**

Our results highlight the burden of the reduction in cancer surgery in 2020 and the risk of delays in diagnosis and treatment for time-dependent conditions. For cancers that can be diagnosed early thanks to screening, the reduction in surgery is likely to be an indirect consequence of discontinuing screening activities. Therefore, further studies are needed to assess, as soon as data are available, the trend in 2021, and to compare our results with those reported in other European countries.

**Key messages:**

• The COVID-19 pandemic caused a significant decline in cancer surgeries in 2020 in Piedmont, Italy. It is necessary to compare our results with those reported in other European countries.

• These results show an increase in the proportion of oncological surgical admissions following emergency access in 2020 compared to the average for 2016-2019.

